# Genotypic Clustering of H5N1 Avian Influenza Viruses in North America Evaluated by Ordination Analysis

**DOI:** 10.3390/v16121818

**Published:** 2024-11-22

**Authors:** Patil Tawidian, Mia K. Torchetti, Mary L. Killian, Kristina Lantz, Krista E. Dilione, Jourdan M. Ringenberg, Sarah N. Bevins, Julianna B. Lenoch, Hon S. Ip

**Affiliations:** 1 U.S. Geological Survey, National Wildlife Health Center Madison, Madison, WI 53711, USA; ptawidian@usgs.gov; 2 Department of Pathobiological Sciences, School of Veterinary Medicine, University of Wisconsin-Madison, Madison, WI 53706, USA; 3 National Veterinary Services Laboratories, U.S. Department of Agriculture, Ames, IA 50010, USA; mia.kim.torchetti@usda.gov (M.K.T.); mary.l.killian@usda.gov (M.L.K.); kristina.lantz@usda.gov (K.L.); 4 Wildlife Service, National Wildlife Disease Program, U.S. Department of Agriculture, Fort Collins, CO 80521, USA; krista.dilione@usda.gov (K.E.D.); jourdan.ringenberg@usda.gov (J.M.R.); sarah.n.bevins@usda.gov (S.N.B.); julianna.b.lenoch@usda.gov (J.B.L.)

**Keywords:** avian influenza, high pathogenic influenza virus (HPAI), H5N1 virus, virus reassortment, H5N1 genotypes, virus nomenclature

## Abstract

The introduction of HPAI H5N1 clade 2.3.4.4b viruses to North America in late 2021 resulted in avian influenza outbreaks in poultry, mortality events in many wild bird species, and spillovers into many mammalian species. Reassortment events with North American low-pathogenic virus were identified as early as February 2022 and over 100 genotypes have been characterized. Such diversity increases the complexity and time required for monitoring virus evolution. Here, we performed ordination and clustering analyses on sequence data from H5N1 viruses identified in North America between January 2020 and December 2023 to visualize the genotypic diversity of viruses in poultry and wildlife populations. Our results reveal that ordination- and cluster-based approaches can complement traditional phylogenetic analyses specifically for the preliminary assignment of H5N1 viruses to genotypic groups or to identify novel genotypes. Our study expands current knowledge on the genotypic diversity of H5N1 viruses in North America and describes a rapid approach for early virus genotype assignment.

## 1. Introduction

Wild migratory waterfowl are well-known reservoir hosts for low pathogenic influenza (LPAI) viruses, either as asymptomatic carriers of the virus or with mild disease [1,2,3]. When H5 or H7 subtype LPAI viruses spillover from the reservoir host into non-host species such as gallinaceous birds (e.g., chickens, turkeys, pheasants) the virus has the potential to mutate to a highly pathogenic form (HPAI); however, spillback into the natural host is rare. The A/goose/Guangdong/1/1996 (Gs/GD) HPAI H5N1 lineage that emerged in poultry, spreading to poultry across other countries, has generated multiple clades (most recently the H5 clade 2.3.4.4) that have spilled back into wild migratory birds resulting in intercontinental transmission across five continents and causing poultry outbreaks, wild bird mortality events, and spillovers into mammalian species [3,4]. This global expansion of the Gs/GD lineage facilitated an increased diversification of the HA gene [5]. 

During the 2020–2021 epidemic wave in Europe, the first report of HPAI H5N1 clade 2.3.4.4b viruses in migratory waterfowl was in the Netherlands during October 2020 [6,7]. Phylogenetic analyses revealed that the newly detected H5N1 virus was a result of a reassortment of a circulating H5N8 2.3.4.4b virus with Eurasian avian lineage LPAI viruses [6]. Since then, H5N1 2.3.4.4b viruses have been detected across numerous countries within Africa, Asia, Europe, North, and South America [5,8]. H5N1 2.3.4.4b viruses, closely related to those in Europe, were detected in North America in December 2021 on the Atlantic coast of Canada [9,10,11]. Time-scaled phylogenetic analyses revealed that the spread of H5N1 to North America was likely due to the movement of migratory waterfowl from Iceland to Canada [11]. The H5N1 clade 2.3.4.4b virus has since disseminated across all four North American flyways with detections in a myriad of wild and domestic bird populations and mammals [12,13,14]. 

Typically, the characterization of genotypic diversity in H5N1 viruses largely relies on phylogenetic analysis and tree construction using gene segments to assess existing and potentially novel genotypes. These analyses and data visualization methods provide useful tools for the study of viral spatiotemporal evolution [9,13]. However, the construction of phylogenetic trees for large datasets can be time consuming and difficult to interpret [15,16]. Ordination analyses are powerful tools for the rapid visualization of evolutionary distances in low-dimensional space [16,17]. Ordination approaches are not a replacement for phylogenetic analysis but, when used in tandem, they provide useful means for efficient and targeted data analysis. Indeed, ordination and phylogenetic analyses have been used in parallel in numerous instances, including, for example, the study of the evolution of the HA and NA genes in influenza A viruses [18], human virome characterization [19], and fungal pathogenesis [20].

The overall goal of this study was to characterize the genotypic expansion of H5N1 2.3.4.4b virus in North America using rapid ordination approaches in concert with phylogenetics. To achieve this goal, we used ordination analyses on distance matrices generated from sequence alignments of over four thousand H5N1 clade 2.3.4.4b viruses detected in North America between its introduction in January 2020 and the end of 2023. Our ordination analyses paralleled the results observed with phylogenetic analyses, revealing the expansion of the A1 genotype into numerous reassortant genotypes upon its circulation in North America. 

## 2. Materials and Methods

### 2.1. Viruses Used in This Study

A total of 4752 H5N1 viruses within the 2.3.4.4b clade, collected between April 2020 and December 2023, were analyzed in this study (Table S1). Viral sequences were obtained from samples submitted to the U.S. Geological Survey (USGS) National Wildlife Health Center (NWHC) and U.S. Department of Agriculture (USDA) National Wildlife Disease Program, which were sequenced at the USDA National Veterinary Services Laboratories (NVSL), and from two data repository banks, Global Initiative for Sharing All Influenza Database (GISAID) EpiFlu [21] and GenBank [22].

#### 2.1.1. NWHC/USDA-NVSL 

From April 2020 to December 2023, 2511 bird and 4 mammal samples were submitted to USDA/NWHC from bird mortality events, hunter-harvest surveillance, and live bird surveillance projects. The 2515 samples used in this study were predominantly waterfowl (*n* = 1143), followed by non-host wild birds referred to as “other wild birds”, including raptors, corvids, and game birds (*n* = 1121), sea/shorebird (*n* = 233), domestic waterfowl (*n* = 12), mammals (*n* = 4), and poultry (*n* = 2). Tracheal/oropharyngeal swabs and cloacal swabs were collected from birds and mammals and tested for avian influenza viruses H5 and 2.3.4.4b H5, as described previously [13]. Samples positive in any of the three tests were sent to NVSL for confirmatory testing including whole-genome sequencing.

#### 2.1.2. GISAID EpiFlu™ Database

To determine the diversity of H5N1 virus genotypes across North America (United States of America and Canada), we further included viruses submitted to GISAID that were identified in birds and mammals from January 2020 to December 2023 (*n* = 2182). These viruses were largely detected in poultry (*n* = 831), followed by waterfowl (*n* = 614), sea/shorebirds (*n* = 122), other wild birds (*n* = 501), domestic waterfowl (*n* = 61), and mammals (*n* = 53). Virus sequences were obtained by downloading the eight gene segments for each viral submission (Tables S1 and S2). For comparison, ten H5N1 virus sequences identified in Europe from GISAID were downloaded and included in the downstream data analyses (Table S1).

#### 2.1.3. GenBank Nucleotide Sequence Database

Additional H5N1 clade 2.3.4.4b virus submissions from January 2020 to December 2023 from the U.S., Canada, and Europe were downloaded from the GenBank nucleotide sequence database and cross-checked for duplicates in GISAID; only unique virus submissions to GenBank were retained (Table S1). The final GenBank dataset included gene segments of 55 H5N1 viruses identified in waterfowl (*n* = 24), other wild birds (*n* = 22), and sea/shorebirds (*n* = 9) (Table S1).

### 2.2. Sequence Pre-Processing

Prior to downstream phylogenetic analyses, sequences from each gene segment were assessed for primer trimming upstream and downstream of the genes’ open reading frames (ORFs). To detect the ORFs and trim the primer sequences, we used the findORFsFasta command in the package ‘ORFik’ [23] adapted to the R Statistical Software (v4.2.2; R Core Team 2022). Each trimmed gene segment was sorted in descending order of size, as follows: PB2, PB1, PA, HA, NP, NA, MP, and NS. Thereafter, downstream phylogenetic and ordination analyses were performed on datasets consisting of either (1) sequences from each gene segment compared across all viruses or (2) concatenated virus genomes consisting of the ORFs in the same gene segment order.

### 2.3. Bioinformatic Data Analyses

#### 2.3.1. Phylogenetic Analysis 

H5N1 viruses were first genotyped using GenoFlu v 1.02 [14]. This tool uses BLAST to identify North American H5NX genomes in the 2.3.4.4b clade from a curated database. Pre-defined genotypes are cross-referenced with the top segment identifications, and a genotype is assigned [24]. A cutoff of 2% difference from the closest curated sequence is used to identify a new reassortment. New reassortments are reviewed using segment-based phylogenetic trees, and new segment sequences added to the curated database as new genotype assignments are identified. At the time of data analysis, H5N1 viruses (*n* = 22) that were not assigned a genotype through the available GenoFLU version were categorized as ‘unknown’ in the downstream analysis. As GenoFlu is continuously being updated, these emerging genotypes may be named in subsequent versions. Multiple sequence alignments on each virus gene segment and concatenated virus genome across all H5N1 viruses were performed using the Multiple Sequence Comparison using the Log- Expectation (MUSCLE) program and the R package ‘msa’ [25]. We then used the Generalized Time Reversible (GTR) substitution model and the discrete Gamma model to describe the rates of evolutionary change through fixed mutations among sequences. We visualized the evolutionary distance among sequences by constructing an ultrafast bootstrap maximum likelihood (ML) phylogenetic tree using the R package ‘phangorn’ [26]. Phylogenetic trees were constructed using the Interactive Tree of Life (iTOL) v6.0 [27]. To enhance the readability of the phylogenetic trees, we have presented the phylogenetic tree associated with gene segment PB2 in two formats: (1) a phylogenetic tree with genotypes annotated on the tree (Figure S2) and (2) a phylogenetic tree without genotype annotation but with host type and flyway (Figure S3). Readers are encouraged to use these trees as a guideline to identify genotypes and associated color scheme for the remaining trees. 

#### 2.3.2. Visualization of Evolutionary Distances Using Clustering Analysis

To allow for a rapid and computationally feasible determination of existing and novel H5N1 virus genotypes, we adopted an ordination-based approach for closely related sequences to accompany phylogenetic trees. We used the R package ‘bios2mds’ [17] to assign Euclidean distance-based difference scores to H5N1 virus sequences. Difference scores were visualized in a low-dimensional space using multidimensional scaling (MDS). To identify genotypic clusters within the ordination analysis, K-means clustering was performed using the base R package “stats (version 3.6.2)”. The ideal number of clusters for the K-means clustering was determined via a silhouette score analysis using the R package ‘bios2mds’. In brief, the silhouette score values ranged from 0.0 to 1.0, where 0.0 is a poor cluster classification and 1.0 is the optimal cluster classification [28]. We then used permutational multivariate analysis of variance (PERMANOVA) with 999 permutations on the distance matrices to determine whether (1) significant differences were detected across host types and bird flyway and (2) if interactions were evident among the identified clusters and each of host type and bird flyway, separately. All statistical analyses and data visualization were performed on R Statistical Software (v4.2.2; R Core Team 2022).

#### 2.3.3. Reference Dataset for H5N1 Virus Ordination and Clustering 

To allow for future H5N1 virus ordination and clustering, we generated a reference dataset consisting of representative H5N1 virus sequences and associated genotypes identified through GenoFLU (Text S1 and Table S3). For genotypes with more than seventy virus assignments, we randomly selected seventy viruses as representatives. All viruses for genotypes with less than seventy virus assignments were included. We also provide an R script (Text S2) that will autonomously perform multiple sequence alignments followed by assignment of Euclidean distance-based difference scores to the sequences, K-means clustering, ordination, and statistical analyses. The R script will require a FASTA file as an input and will ouput a csv file with the virus identifiers and the K-means cluster associated with each virus. While detailed instructions are provided, a foundational knowledge of the R Statistical Software is required to effectively implement the analyses described in this study.

## 3. Results

### 3.1. Introduction of H5N1 Viruses to North America

Our analysis of 4752 H5N1 viruses within the 2.3.4.4b clade and their gene segments revealed 5 H5N1 viruses introduced into North America. The earliest introduction (A1 genotype), through the Atlantic flyway, was detected in the United States of America (USA) on 30 December 2021 and last detected in our dataset on 28 November 2022. The A1 genotype accounted for 10.5% of the total H5N1 viruses (*n* = 500) detected in North America and was primarily associated with waterfowl (41.0%) followed by poultry (37.4%), other wild birds (14.0%), sea/shorebirds (6.0%), and domestic waterfowl (1.60%) (Table S1). On May 2022, a second introduction event was detected from the Atlantic flyway into the USA known as the A2 genotype. The A2 genotype represents 3.9% of all H5N1 viruses (*n* = 186) detected in our dataset between 16 February 2022 and 30 September 2023. In contrast to the A1 genotype, viruses within the A2 genotype were detected mostly in sea/shorebirds (45.2%) rather than waterfowl (22.0%) followed by other wild birds (21.5%) and poultry (3.8%). The A2 genotype was the only non-reassorted genotype among this dataset detected in mammals (7.5%) (Table S1). The third introduction was through the Pacific flyway (A3) and was first detected in Canada on 3 February 2022 in a deceased bald eagle (*Haliaeetus leucocephalus*). It was then detected in the USA on 26 April 2022 and last detected in this dataset on 19 September 2023. This genotype accounted for 2.0% of the total North American H5N1 viruses (*n* = 96) in our dataset and was largely identified in other wild birds (59.4%) (Table S1). The fourth introduction (A4) was identified in waterfowl within Alaska, USA, during October 2022. This genotype accounted for 0.1% (*n* = 6) of the total H5N1 viruses identified in this study (Table S1). The most recent introduction in our dataset (A5) accounted for 0.2% (*n* = 8) of the total viruses and was introduced into North America via the Atlantic flyway in sea/shorebirds. 

### 3.2. Ordination Analysis Recapitulates H5N1 Genotypic Diversity Observed by Phylogentic Analysis 

**Figure 1:**
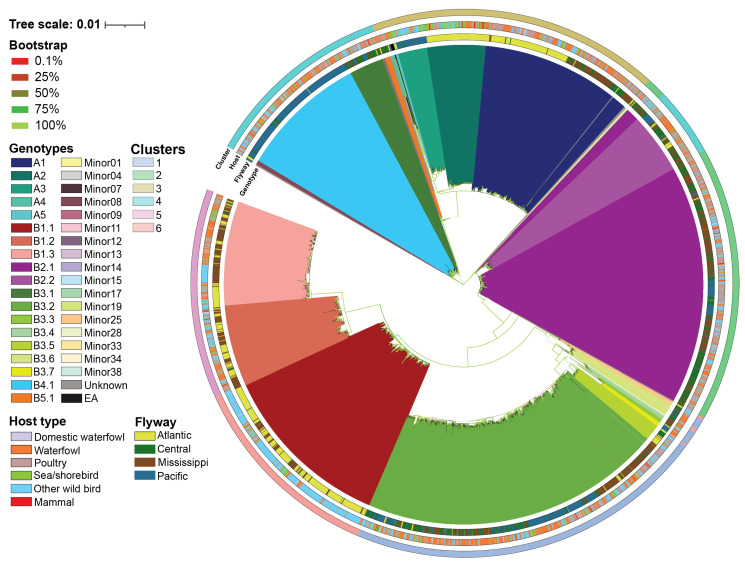
Maximum likelihood phylogenetic tree with ultrafast bootstraps of whole genome of H5N1 2.3.4.4b viruses detected in North America between April 2020 and December 2023. Host and flyway for each virus are assigned as a color-coded ring on the outer edge of the phylogenetic tree. Color keys of genotypes, ultrafast bootstraps, K-means cluster, host category, and flyway are assigned at the left of the figure.

**Figure 2:**
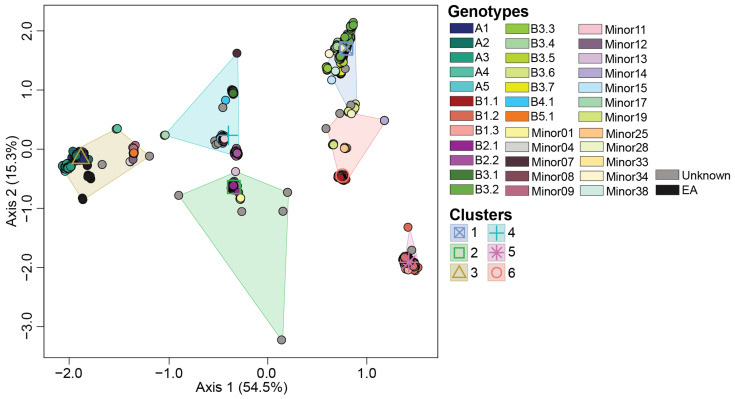
Multidimensional scaling to visualize the Euclidean distances computed on the multiple sequence alignment of whole genome of H5N1 viruses detected in North America. Centroid of each K-means cluster is depicted in an outlined shape. Genotypes and groups are color coded per the color key at the right portion of the figure.

A total of 36 H5N1 genotypes were assigned to our dataset using GenoFLU. This agreed with a maximum likelihood phylogenetic analysis conducted on the multiple sequence alignment of the whole genome of H5N1 viruses (Figure 1). To assess whether the genotypic diversity observed using the phylogenetic analyses was recapitulated with the ordination-based approach, we computed the Euclidean distances of the full ORF genome of H5N1 viruses’ multiple sequence alignments. Our ordination analysis and the subsequent K-means clustering supported the genotypic diversity observed in the phylogenetic analysis, including five virus introductions to North America (genotypes A1, A2, A3, A4, and A5). Six distinct clusters (Silhouette score: 0.797) were identified across the 36 genotypes in our dataset (Figure 2, Table 1). The largest cluster was cluster 1 accounting for 23.5% of viruses identified by GenoFLU followed by clusters 2 (17.9%), 3 (17.7%), 4 (15.7%), 5 (12.8%), and 6 (12.5%). The A genotypes (not reassorted) clustered together in cluster 3 along with the ten European reference viruses. Cluster 3 also contained several minor genotypes as well as genotype B5.1 (Table 1). Other reassortant genotypes created distinct clusters. In addition, the ordination and cluster analyses assigned clusters to the viruses that were unassigned by GenoFLU (Figure 2), which was not unexpected but further supports the usefulness of an ordination-based tool for data visualization, especially when identifying new genotypes. Unidentified viruses by GenoFLU were detected across all clusters: clusters 2 and 3 (*n* = 6 each), cluster 4 (*n* = 4), cluster 1 (*n* = 3), cluster 6 (*n* = 2), and cluster 5 (*n* = 1) (Table S1). Some of these viruses (*n* = 8) were detected in Canada while others were detected in the USA.

The observed clusters were significantly different from one another, as supported by a PERMANOVA analysis (pseudo-F = 70.3; *p* < 0.001), indicating distinct clustering patterns. The ordination-based approach allows for a rapid, albeit lower resolution, cluster identification of H5N1 viruses in less than two hours using a standard laptop, compared to the phylogenetic approach with ultrafast bootstrapping conducted in this study, which took up to five days using a high-throughput computing environment. Thus, the cluster-based approach was a robust and effective alternative for traditional phylogenetic analysis for the preliminary H5N1 virus classification and visualization into genotype clusters.

### 3.3. Reassortant H5N1 Virus Genotypes

Our dataset contains 31 reassortant genotypes that were associated with the A1 genotype. Reassortment events associated with the A2, A3, A4, and A5 genotypes were not detected in this dataset. The majority of reassortant genotypes were collected during early 2022 and continued to circulate until December 2023. Fourteen reassortant genotypes were assigned to the group “B” while 17 were classified as “Minor” genotypes. 

Within the reassortant genotype “B” viruses, genotypes B1, B2, B3, B4, and B5, were classified. The most common reassortant genotype was B3.2 which accounted for 25.3% of all of the reassortant H5N1 virus genotypes. Genotype B3.2 was first detected in a bald eagle collected from the Mississippi flyway on 20 March 2022. Thereafter, it was detected largely in waterfowl (48.1%) and other wild birds (26.1%) (Table S1). The second most common reassortant genotype, B2.1, accounted for 21.3% of the reassortant genotypes. This genotype was first detected on 1 March 2022 in a bald eagle from the Mississippi flyway (Table S1). This genotype was primarily detected in other wild birds (32.9%) and waterfowl (31.9%). Genotype B1.1 was first detected in a bald eagle sample collected from the Atlantic flyway on 25 January 2022 (Table S1). This genotype represents the third most common reassortant (14.6%) and was largely associated with other wild birds (79.6%). Of the remaining genotypes, B4.1 was detected in a poultry sample in Canada during 5 March 2022 through the Pacific flyway. It was then detected in the USA starting 4 April 2022 (Table S1). This genotype accounted for 10.7% of reassortant genotypes. The least common genotype among this dataset was B5.1 (0.5%) and it was primarily detected in waterfowl starting from 1 March 2022 (Table S1). 

The genotypes assigned to the groups “Minor”, in sum, accounted for 1.5% of the reassortant genotypes detected in North America. The most common genotypes were Minor19 (*n* = 9), Minor01 and Minor08 (*n* = 8 each), Minor07 (*n* = 7), Minor09 (*n* = 5), and Minor14 (*n* = 4) (Table S1). While the remaining genotypes are mostly detected in one or two hosts and, as of the publication of this study, remain sporadic. 

### 3.4. H5N1 Virus Reassortants Were Limited to Five Viral Gene Segments

**Figure 3:**
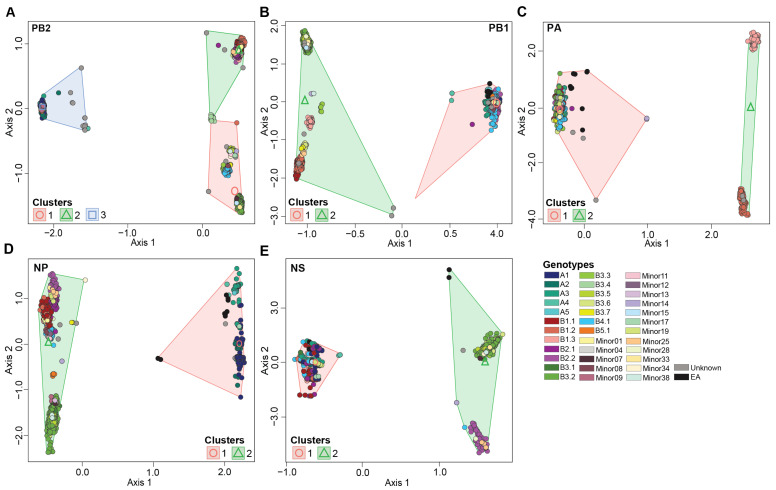
Multidimensional scaling to visualize the dissimilarity distances computed on the multiple sequence alignment of H5N1 virus gene segments. (**A**) Axes 1 and 2 of gene segment PB2; (**B**) axes 1 and 2 of gene segment PB1; (**C**) axes 1 and 2 of gene segment PA; (**D**) axes 1 and 2 of gene segment NP; (**E**) Axes 1 and 2 of gene segment NS. Genotypes and K-means clusters are color coded per the color key at the bottom right of the figure.

To identify gene segments involved in H5N1 virus reassortment, we performed ordination-based approaches on the reassorted gene segments (Figure 3 and Figure S1). In addition, we determined the patterns observed to those seen by the maximum likelihood phylogenetic analysis on each individual gene segment. H5N1 reassortment events among this dataset involved five gene segments: PB2, PB1, PA, NP, and NS (Figures S2–S7). Reassortment events were not detected in the gene segments HA, NA, or MP (Figures S8–S10). 

#### 3.4.1. PB2 Gene Segment

The PB2 gene segment had three K-mean clusters (1, 2, and 3) across the H5N1 viruses in this study (Figure 3A). PB2-clusters 1 and 2 were composed of reassortant viruses (*n* = 3912; 82.3%) while PB2-cluster 3 was primarily composed of introduced genotypes. PB2-clusters 1 and 2 contain genotypes with a North American PB2 gene while PB2-cluster 3 contains genotypes with a Eurasian PB2 gene. PB2-cluster 1 accounted for most genotypes identified in this study (*n* = 19). Members within this cluster included viruses belonging to the B4.1 genotypes and the majority of B3 genotypes, except for some B3.4 and all B3.6 viruses, and nine minor genotypes (Minors 07, 08, 11, 14, 15, 17, 33, 34, and 38) (Figure 3A). The second largest cluster was PB2-cluster 2 (*n* = 13) encompassing all viruses within the genotypes B1, B2, B3.4, B3.6, and Minors 01, 13, 19, 25, and 28. PB2-cluster 3 was made up of A genotypes, B5.1, and three minor genotypes, Minors 04, 09, 12. PB2-cluster 3 was the most divergent cluster compared to 1 and 2.

To further investigate the genotypic diversity within PB2-clusters 1 and 2, we performed ordination analysis and K-mean clustering on a dataset without members of PB2-cluster 3 (Figure S1A). Our analysis reveals three novel reassortant clusters, which assign genotypes B3.5 and B4.1 to a distinct cluster separate from the B1/B2 and B3 genotypes. The patterns observed in our ordination assay were recapitulated in the phylogenetic tree associated with the PB2 gene segment with 100% bootstrap support (Figures S2 and S3).

#### 3.4.2. PB1 Gene Segment 

A phylogenetic analysis conducted on segment PB1 revealed four main clusters, one cluster containing the A viruses and three reassortant clusters (*n* = 2319; 48.8%) (Figure S4). Similar patterns were observed in the ordination analyses (Figure 3B and Figure S1B). An ordination analysis on the entire dataset revealed two significantly different clusters, PB1-cluster 1 contains genotypes with a Eurasian PB1 gene (genotypes A, B2s, B3.1, B4.1, B5.1, and numerous minor genotypes). Whereas PB1-cluster 2 viruses contain a North American PB1 gene segment, namely B1 and B3 genotypes (Figure 3B). In turn, an ordination analysis performed on the genotypes within PB1-cluster 2, reveals further separation of reassortant genotypes into three sub-clusters (Figure S1B). PB1-cluster 2a was primarily associated with genotypes B1.3 and B3.3, while PB1-clusters 2b and 2c were associated with genotypes B1.1/B1.2 and B3s, respectively (Figure S1B). 

#### 3.4.3. PA Gene Segment

Ordination and phylogenetic analyses revealed three clusters along the PA gene segment (Figure 3C and Figure S5). PA-cluster 1 was the largest cluster and contained the Eurasian not reassorted viruses and those retaining the Eurasian PA gene segment, while PA-cluster 2 was associated with the reassortant genotypes B1.2 and B1.3 representing a North American PA gene (Figure 3C). In addition, the ordination analysis performed on genotypes B1.2 and B1.3, reveals distinct clustering patterns across the two genotypes (Figure S1C). 

#### 3.4.4. NP Gene Segment

The gene segment NP had the highest number of reassortant viruses (*n* = 3937; 82.8%) in addition to the original non-reassorted cluster (Figure S6). The ordination analysis revealed two main clusters separating the original and reassortant genotypes (Figure 3D). NP-cluster 1 was associated with the Eurasian non-reassorted viruses and Minor 17, while NP-cluster 2 consisted of all B genotypes and most minor genotypes. In turn, the ordination analysis performed on the viruses within NP-cluster 2 identified four significantly different sub-clusters. These sub-clusters showed distinct clustering patterns of reassortant viruses that belonged to genotypes B1, B2, B3, B4, and B5 (Figure S1D). 

#### 3.4.5. NS Gene Segment

Two reassortant clusters (*n* = 1320, 27.8%) were identified in gene segment NS in addition to the non-reassorted cluster which accounted for the largest virus assignment (Figure 3E and Figure S7). NS-cluster 1 included the Eurasian non-reassorted genotypes while NS-cluster 2 included the reassortant genotypes B2.2, B3.2, B3.3, B3.4, B3.5, B3.6, B3.7, and seven minors (Figure 3E). In turn, genotype B2.2, Minors 14, 25, and 28 had significantly different clustering patterns than the remaining reassortant genotypes (Figure S1E). 

### 3.5. Migratory Flyway and Host Type May Impact Viral Genotypic Diversity

**Figure 4:**
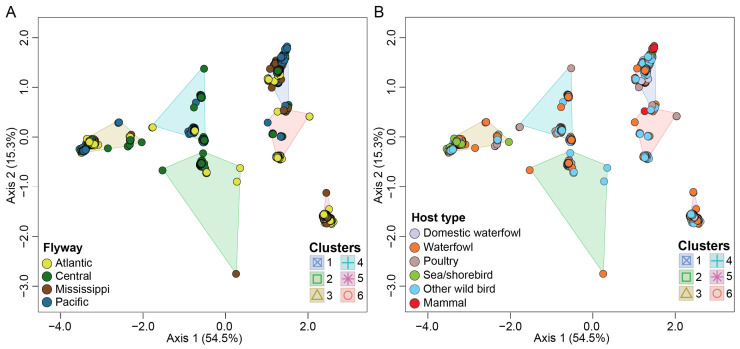
Multidimensional scaling to visualize genotypic diversity of H5N1 viruses in North America. (**A**) Flyway; (**B**) host type. Each variable is color coded differently as represented by the color key at the bottom left of each panel.

Our results revealed a significant impact of bird flyway on H5N1 genotypic diversity in North America (pseudo-F = 32.2; *p* < 0.001, R^2^ = 0.100) (Figure 4A). Our data show that the number of identified H5N1 viruses and their associated genotypes vary across subregions within individual bird flyways across the USA. For example, the Mississippi flyway accounted for the highest number of identified H5N1 viruses within North America (*n* = 1483; 31.3%). However, more than 80% of these viruses (*n* = 1223) were identified only in states within the midwestern region of this flyway compared to the northeastern, southeastern, and southcentral regions. Similar results were observed for the Central flyway (*n* = 937; 19.8%) where the northcentral regions accounted for the highest number of viruses (*n* = 597; 63.6%) identified within this flyway. Furthermore, several H5N1 virus clusters were specific to a subregion within a flyway as compared to other regions, as supported by a significant interaction (pseudo-F = 6.42; *p* < 0.0001; R^2^ = 0.046) between H5N1 genotypic clusters and bird flyway. In addition, a significant interaction was detected between host type and flyway (pseudo-F = 13.1; *p* < 0,001; R^2^ = 0.037).

Finally, we show differences in H5N1 virus distribution across host type (pseudo-F = 45.5; *p* < 0.001; R^2^ = 0.042) (Figure 4B). As may be expected, the majority of H5N1 viruses analyzed in this study were identified in waterfowl (37.5%) and other wild birds (34.6%). 

### 3.6. Reference Dataset Is Robust Enough to Be Used for Genotype Assignment of Newly Sequenced H5N1 Viruses Using Ordination Analysis

**Figure 5:**
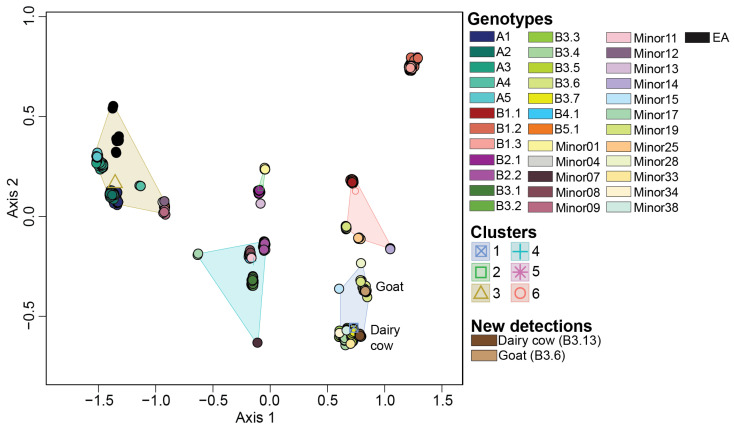
Multidimensional scaling performed on the dissimilarity distances on the viruses within the reference dataset. The centroid of each K-means clusters is depicted as an outlined shape. Genotypes and clusters are color coded per the color key at the right portion of the figure. Novel viruses downloaded from GISAID are categorized under “New detections” and annotated on the MDS plot.

To allow for the rapid genotypic assignment of newly detected H5N1 viruses in North America, we generated a reference dataset containing 983 viruses spanning all genotypes identified in this study. We ensured that the reference dataset recapitulates the genotypic clusters observed in the original dataset by performing ordination analysis on the dissimilarity distances followed by K-means clustering. We further validated this dataset by including recently detected H5N1 viruses from dairy cows (*n* = 17) collected from Ohio, South Dakota, and Texas and goats (*n* = 6) from Minnesota (Figure 5). As observed in the original dataset, six K-means clusters were detected in the reference dataset. Each cluster was associated with the same genotypes as the original dataset (Figure 5). The dairy cow and goat samples were identified within cluster 1, which was associated with the majority of B3 genotypes, and consistent with expectations based upon phylogeny. All dairy cow samples clustered closely with genotype B3.7, whereas the goat samples clustered with genotype B3.6 as expected as this virus was identical to what the chickens and ducks on the same premises had. The virus affecting dairy cows is genotype B3.13. Ordination analysis performed on genotypes B3.6, B3.7, and B3.13, reveal distinct clustering patterns across B3.7 and B3.13 genotypes (Figure S11). These results demonstrate that use of the reference dataset generated by this study allows for the rapid grouping of new viruses based upon ordination.

## 4. Discussion

Multiple genotyping schemes have been developed for A/goose/Guangdong/1/1996 (Gs/GD) H5N1 lineage viruses. Because of the complex circulation of these viruses within flyways, the distinct genotyping schemes utilized in Asia [29], Europe [7], and North America [14] have utility for better understanding and monitoring the changes in and movement of these viruses within those geographic areas. The GenoFLU tool used in this study was developed to characterize introductions of the 2.3.4.4b clade to the USA as well as subsequent reassortments of that virus with low-pathogenic North American viruses [14]. This study aimed at implementing strategies for the efficient detection and visualization of H5N1 2.3.4.4b virus genotypic diversity across North America. To this end, we used a multidimensional scaling (MDS) analysis as an alternative to phylogenetic analysis for the identification of reassortant H5N1 viruses across North America between January 2020 and December 2023. Our results revealed that the MDS approach readily paralleled the results observed with phylogenetic analysis while reducing time and computational demands. To our knowledge, this is the first study that uses a clustering approach in tandem with phylogenetic analyses for the genotypic assignment of H5N1 viruses in North America. Ordination-based approaches are used in addition to phylogenetic analyses in numerous host–virus systems, including the study of coronavirus evolution through the analysis of the spike protein [30]. Similarly, studies that assess the virome associated with human blood and plasma utilize cluster-based methods to show the evolutionary space of anelloviruses in relation to viruses within reference databases [19,31]. This approach is not limited to the study of viral evolution in mammalian systems but is also used in the identification of tick evolution across various geographical spans [32]. While ordination analysis effectively detects clustering patterns in a dataset, it has some drawbacks, including sensitivity to outliers, potential for overfitting, and loss of information through masking smaller yet meaningful variations. Thus, the utilization of MDS approaches in addition to phylogenetic analyses can provide a powerful compendium of tools to study evolutionary changes in targeted populations within and across host systems.

The dataset used in this study identified five distinct H5N1 2.3.4.4b virus introductions in North America [14], three of which arrived through the Atlantic flyway (A1, A2, and A5) [9,10] and two via the Pacific flyway (A3 and A4) [33]. Interestingly, our analysis of viruses not assigned genotypes by GenoFLU revealed viruses that clustered with the A1 genotype which were first detected in Canada in mid-December 2021 where the A1 genotype was then detected in the USA in late December 2021. While some viruses collected from Canada were not assigned to any genotype, the clustering pattern supports the circulation of the A1 genotype in Canada prior to the USA [34,35]. Similarly, the A3 genotype was first detected in a bald eagle sample in British Columbia, Canada, collected in February 2022, followed by Alaska, USA, in April 2022. The remaining A genotypes were only detected in the USA across 2022 and 2023 [14,36]. We acknowledge that virus sequences from Canada were under-represented in our study, such as the H5N5 (A6 genotype) that has been found in Atlantic Canada and in the northern Atlantic flyway in the USA at the beginning of 2023 [37]. As such, we cannot exclude the possibility that the reassortant genotypes A2, A4, A5 or A1 were circulating in Canada prior to their detection in the USA. Waterfowl migratory flyways span North America in a north–south orientation [38], making it likely that common H5N1 genotypes in the United States also occur in Canada. Indeed, a previous study reports the identification of a reassortant H5N1 genotype containing gene segments closely related to the North American A1 genotype in broiler chicken in Canada [39], revealing the potential circulation of reassortant H5N1 genotypes in Canadian regions. Thus, future efforts to obtain historical H5N1 virus sequences from Canada would be beneficial to better understand the introduction and emergence of reassortant H5N1 2.3.4.4b viruses in North America. In addition, while over a thousand H5N1 genomes from the year 2022 introduction into North America have been made available, further sequencing of yet untested samples may reveal additional introductions, such as the H5N5 [40], and help elucidate the evolutionary history of these viruses following their introduction.

Reassortment events within H5N1 2.3.4.4b viruses are not surprising and have been reported across numerous continents [8,29]. Indeed, studies provide evidence of numerous reassortment events involving one or more gene segments of the virus. For example, a recent study characterized 16 distinct reassortant virus genotypes identified in 233 H5N1 2.3.4.4b virus sequences across countries within Africa, the North Americas, Asia, and Europe [29,41]. While one genotype classified as G1, based on the Asian classification scheme [29], was widely spread across countries from all four continents, numerous other genotypes were associated with specific geographic locales. Furthermore, a recent study of genotypic diversity of H5N1 virus in the USA, revealed twenty-one distinct clusters in which six reassortant genotypes accounted for 92% of all of the viruses identified in the USA [14]. Our results parallel those described by Youk et al., [14] for North America, supporting the emergence of reassortant genotypes from wild bird LPAI viruses. 

Our study identified that the reassortment events were associated with five gene segments of H5N1 viruses. These data were supported by studies that report reassortment events associated with the same gene segments across the USA and other countries [14,42,43]. Reassortment events associated with avian influenza viruses are shown to generate significant viral diversity and facilitate host specificity and virulence [44] and may have contributed to the rapid spread of influenza viruses and their emergence in novel hosts [5]. Our data curation using public repositories revealed numerous examples of H5N1 viruses detected in North America from mammalian hosts, such as bobcats (*Lynx rufus*), red foxes (*Vulpes vulpes*), bottlenose dolphins (*Tursiops truncatus*), skunks, and seals. A similar host expansion is increasingly reported across North America, with novel detections in marine mammals including dolphins [45] and a recent spillover into dairy cows [46,47,48] with an associated increase in human detections [46]. Interestingly, in most cases, viruses detected in mammals were associated with reassortant genotypes, especially genotypes with PB2 reassortment. In addition, reassortant H5N1 2.3.4.4b viruses previously detected in North America were also detected in marine mammals in Peru [42]. 

The circulation of LPAI H5N1 in poultry populations allowed for the initial emergence of the HPAI H5N1 goose/Guangdong lineage viruses and their spillover to wild bird populations [49,50]. Following their introduction to the United States, the wide distribution of HPAI viruses in wild waterfowl species that are natural hosts for North American LPAI viruses created the potential for reassortment events and the emergence of novel genotypes [51]. While some mammalian hosts were detected, our study found that most H5N1 HPAI viruses in North America during this period were associated with avian hosts, particularly waterfowl and other wild birds. In addition, we showed varied H5N1 virus clustering patterns, as measured by host type. For example, genotype B4.1 was mostly detected in waterfowl followed by other wild birds. In addition, several reassortant genotypes were identified in mammals but were, in turn, not identified within the A1 genotype. This dataset suggests an association of viral genotypic diversity with host species; however, genotype success or frequency as well as potential sampling biases in the analyzed dataset, are acknowledged. For example, (1) while genotype B1.1 was associated with other wild birds, this genotype was largely associated with black vulture (*Coragyps atratus*) mortality events (*n* = 350/456). These animals roost together and are known to feed on other members of the roost, perpetuating the infection [52]. (2) Bald eagles are large birds with high visibility, potentially increasing the sampling of bald eagle samples within mortality events of raptors. And (3), there was a limited surveillance in mammals.

We also show that avian migratory flyways and sub-regions within a flyway may further contribute to H5N1 genotypic diversity in North America. Four introductions of H5N1 to North America were limited to the Atlantic flyway, while two introductions were limited to the Pacific flyway. However, reassortant genotypes had a higher rate of detection within subregions of the Central and Mississippi flyways. Our results agree with previous reports of genotypic diversity associated with geographic ranges, including bird flyways [14,53]. This result may reveal a geographic signature of H5N1 virus detection; however, some degree of sampling bias and data heterogeneity may also affect the magnitude of the effect size and the observed distribution of genotypes. While surveillance mechanisms exist for monitoring avian influenza in wild migratory waterfowl, the absence of a targeted and consistent surveillance strategy for use in other wild bird species and mammals provides an incomplete understanding of the full transmission dynamics and the distribution of different genotypes.

In conclusion, our study reveals that ordination and cluster-based approaches can complement traditional phylogenetic analyses specifically for the preliminary assignment of H5N1 viruses to genotypic groups or to identify novel genotypes. A rapid genotypic assignment of newly detected H5N1 viruses was achieved using a reference dataset that recapitulates the genotypic clusters followed by K-means clustering. Using this approach, we provide a fundamental insight into the genotypic diversity and spread of H5N1 2.3.4.4b viruses across the USA which expands current knowledge on the genotype diversity within H5N1 viruses in North America. This work also highlights the need for more widespread and standardized surveillance strategies for the accurate depiction of reassortant genotype circulation within non-target host populations, including mammalian hosts. 

## Figures and Tables

**Table 1:** Summary of detection dates, flyways, and K-means cluster assignments of viruses that also have a genotype assigned by GenoFLU.

Cluster	Genotype	First Detected	Last Detected	Flyways (N Viruses)				Total Viruses
				Atlantic	Central	Mississippi	Pacific	
1	B3.2	20 March 2022	22 December 2023	40	261	348	343	1111
	B3.3	8 October 2022	6 April 2023	3	2	2	x	
	B3.4	23 November 2022	7 March 2023	x	2	15	x	
	B3.5	30 October 2022	18 May 2023	25	17	4	x	
	B3.6	22 November 2022	12 October 2023	x	13	6	12	
	B3.7	11 September 2023	11 October 2023	x	1	x	12	
	Minor15	29 April 2022		x	x	1	x	
	Minor33	21 February 2023		1	x	x	x	
	Minor34	17 September 2022		x	x	1	x	
	Minor38	18 October 2022		2	x	x	x	
2	B2.1	1 March 2022	7 September 2023	5	334	396	99	843
	Minor01	18 February 2022	27 April 2022	5	x	3	x	
	Minor13	15 March 2022		x	1	x	x	
3	A1	30 December 2021	28 November 2022	263 *	74	162	1	824
	A2	16 February 2022	30 September 2023	186 *	x	x	x	
	A3	3 February 2022	19 September 2023	x	6	1	89 *	
	A4	14 October 2022		x	x	x	6 *	
	A5	31 October 2022	3 February 2023	8 *	x	x	x	
	B5.1	1 March 2022	7 April 2022	x	13	6	x	
	Minor04	9 April 2022		x	2	x	x	
	Minor09	1 February 2022	1 March 2022	4	x	1	x	
	Minor12	1 April 2022		x	2	x	x	
4	B2.2	28 March 2022	20 April 2023	22	78	59	38	742
	B3.1	4 March 2022	1 November 2022	2	65	24	23	
	B4.1	5 March 2022	11 April 2023	1	28	4	385	
	Minor07	26 April 2022		x	1	x	x	
	Minor08	1 February 2022	25 April 2022	8	x	x	x	
	Minor11	1 April 2022	4 April 2022	x	2	x	x	
	Minor17	1 November 2022		2	x	x	x	
5	B1.2	12 February 2022	6 March 2023	124	6	131	x	605
	B1.3	26 June 2022	28 April 2023	130	7	207	x	
6	B1.1	25 January 2022	6 April 2023	460	4	107	4	596
	Minor14	22 March 2022	25 March 2022	4	x	x	x	
	Minor19	18 November 2022	9 January 2023	x	7	2	x	
	Minor25	22 November 2022	13 December 2022	x	5	1	1	
	Minor28	21 November 2022		x	x	1	x	

* Refers to flyway where this genotype was first detected in the USA; “x” indicates that no virus from our dataset was collected from the corresponding flyway.

## Data Availability

The data presented in this study are available in GISAID and NCBI GenBank. Those uploaded to NCBI for this manuscript are available associated with BioProject PRJNA1186741. These data were derived from the following resources available in the public domain: GISAID (http://www.gisaid.org) and NCBI (https://www.ncbi.nlm.nih.gov).

## Supplementary Materials

The following supporting information can be downloaded at: https://www.mdpi.com/article/10.3390/v16121818/s1. Figure S1: Multidimensional scaling to visualize genotypic diversity of reassortant H5N1 viruses associated with each gene segment. (A) Axes 1 and 2 of gene segment PB2; (B) Axes 1 and 2 of gene segment PB1; (C) Axes 1 and 2 of gene segment PA; (D) Axes 1 and 2 of gene segment NP; (E) Axes 1 and 2 of gene segment NS. Genotypes and K-means clusters are color coded per the color key at the bottom right of the figure. Figure S2: Phylogenetic tree with ultrafast bootstraps of PB2 gene segment. Genotypes, flyway, and host category are color coded differently and bootstrap support values are indicated categorically. Figure S3: Phylogenetic tree with ultrafast bootstraps of PB2 gene segment with genotypes annotation on graph. Genotypes are color coded differently and bootstrap support values are indicated categorically. Figure S4: Phylogenetic tree with ultrafast bootstraps of PB1 gene segment. Genotypes, flyway, and host category are color coded differently and bootstrap support values are indicated categorically. Figure S5: Phylogenetic tree with ultrafast bootstraps of PA gene segment. Genotypes, flyway, and host category are color coded differently and bootstrap support values are indicated categorically. Figure S6: Phylogenetic tree with ultrafast bootstraps of NP gene segment. Genotypes, flyway, and host category are color coded differently and bootstrap support values are indicated categorically. Figure S7: Phylogenetic tree with ultrafast bootstraps of NS gene segment. Genotypes, flyway, and host category are color coded differently and bootstrap support values are indicated categorically. Figure S8: Phylogenetic tree with ultrafast bootstraps of HA gene segment. Genotypes, flyway, and host category are color coded differently and bootstrap support values are indicated categorically. Figure S9: Phylogenetic tree with ultrafast bootstraps of NA gene segment. Genotypes, flyway, and host category are color coded differently and bootstrap support values are indicated categorically. Figure S10: Phylogenetic tree with ultrafast bootstraps of MP gene segment. Genotypes, flyway, and host category are color coded differently and bootstrap support values are indicated categorically. Figure S11: Multidimensional scaling to visualize genotypic diversity of reassortant H5N1 whole-genome viruses belonging to the genotypes B3.6, B3.7, and B3.13. Table S1: Genotypic assignment of each virus detected in North America with description of collection date, host species, and bird flyway. Table S2: Accession numbers for H5N1 sequences from different repositories used in this study. Table S3: Genotypic assignment of each virus used in the reference dataset. Text S1: FASTA format file containing the reference HPAI H5N1 virus sequences. Text S2: R Script for Bioinformatics Analysis of Avian Influenza Virus Data.
