# Supplementary material for: Genotypic Clustering of H5N1 Avian Influenza Viruses in North America Evaluated by Ordination Analysis
Source: Viruses. 2024 Nov 22;16(12):1818. doi: 10.3390/v16121818 (PMC11680268; doi:10.3390/v16121818)
Supplement: Supplementary file 1 [file viruses-16-01818-s001.zip › Supplementary Figures.pdf]

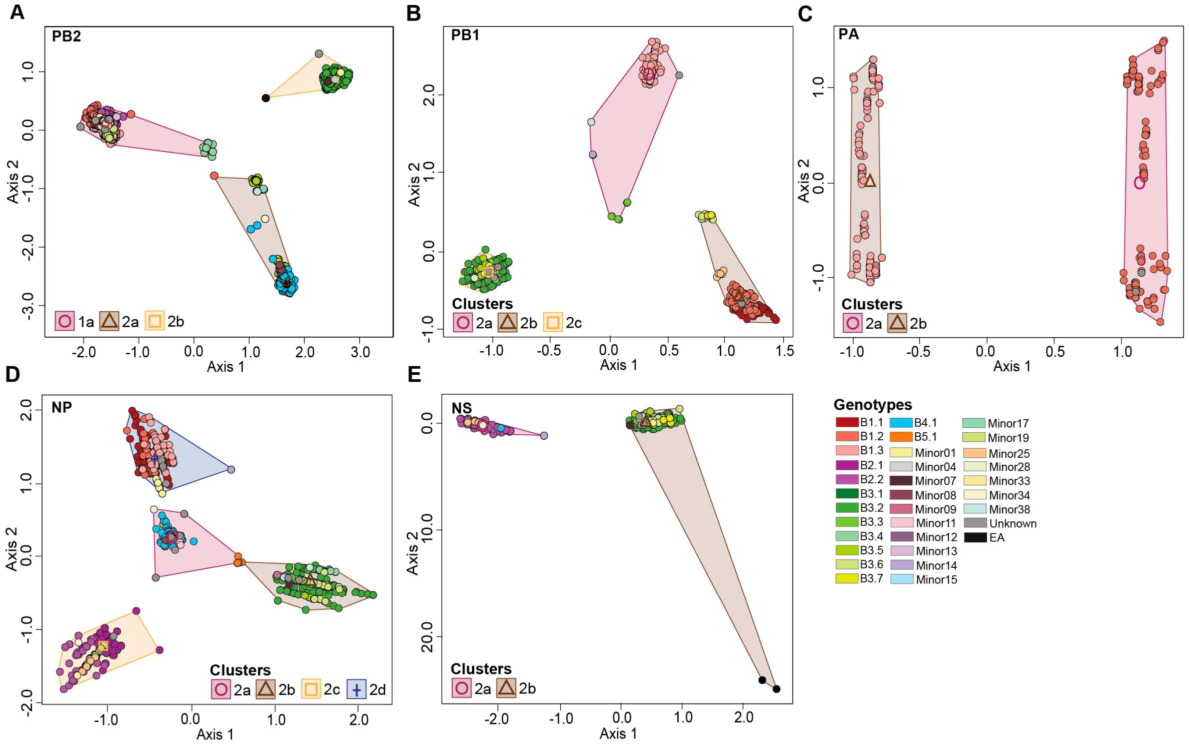

Figure S1. Multidimensional scaling to visualize genotypic diversity of reassortant H5N1 viruses associated with each gene segment. (A) Axes 1 and 2 of gene segment PB2; (B) Axes 1 and 2 of gene segment PB1; (C) Axes 1 and 2 of gene segment PA; (D) Axes 1 and 2 of gene segment NP; (E) Axes 1 and 2 of gene segment NS. Genotypes and K-means clusters are color coded per the color key at the bottom right of the figure.

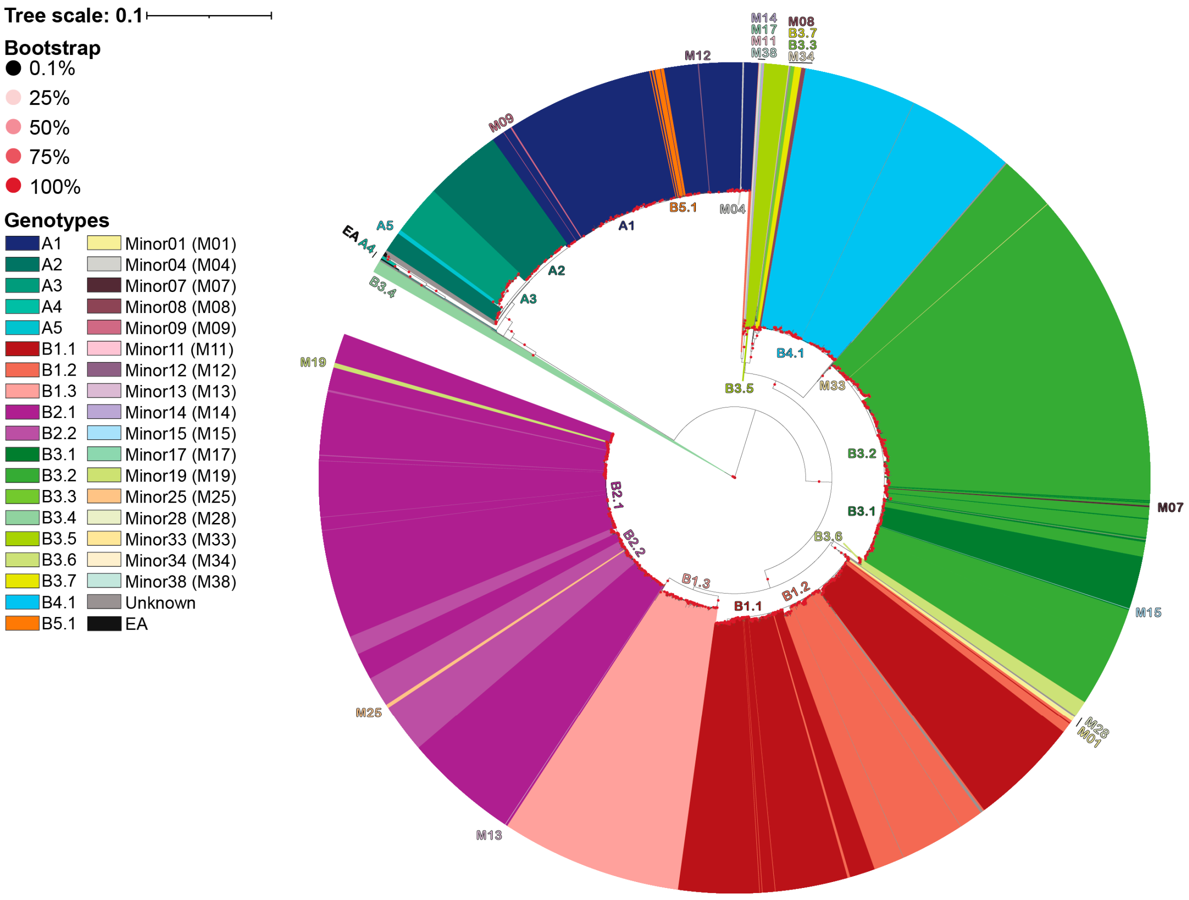

Figure S2. Phylogenetic tree with ultrafast bootstraps of PB2 gene segment. Genotypes, flyway, and host category are color coded differently and bootstrap support values are indicated categorically.

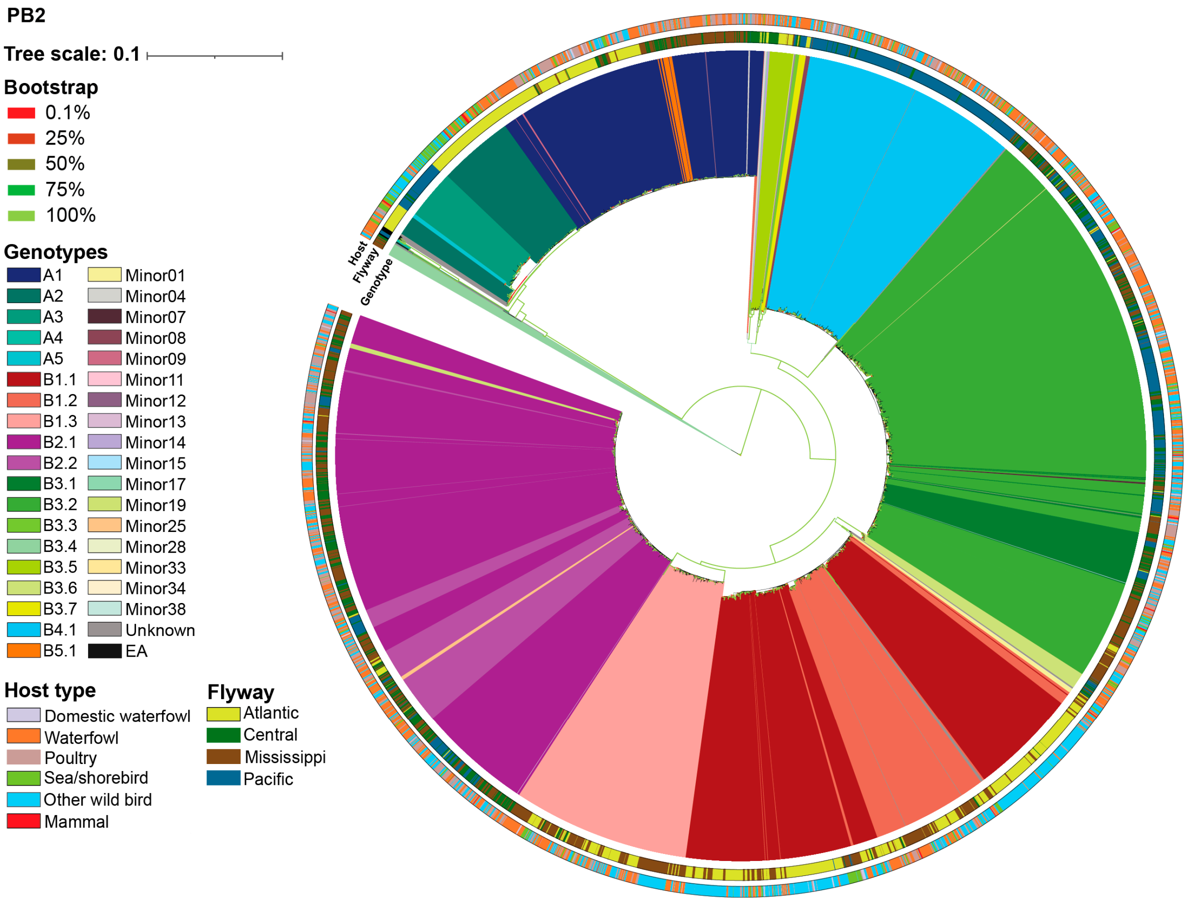

Figure S3. Phylogenetic tree with ultrafast bootstraps of PB2 gene segment with genotypes annotation on graph. Genotypes are color coded differently and bootstrap support values are indicated categorically.

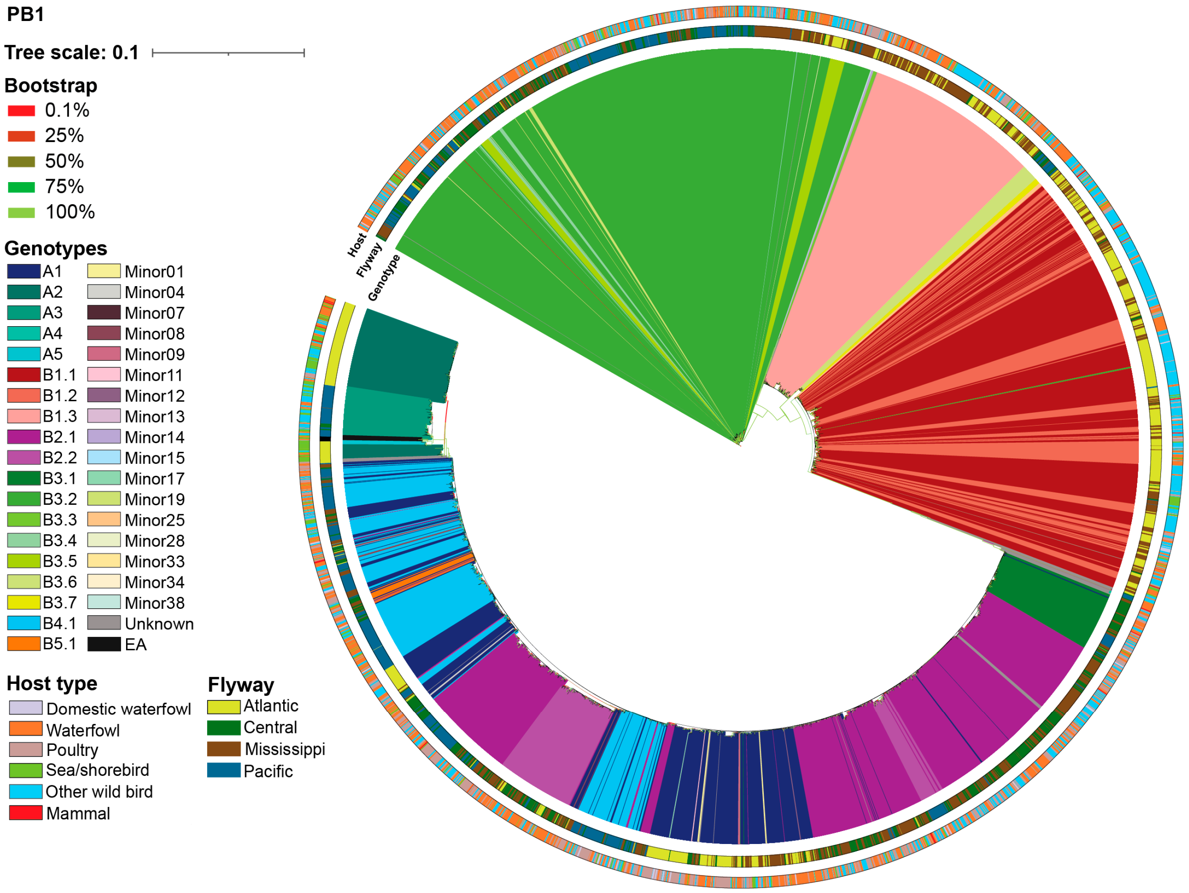

Figure S4. Phylogenetic tree with ultrafast bootstraps of PB1 gene segment. Genotypes, flyway, and host category are color coded differently and bootstrap support values are indicated categorically.

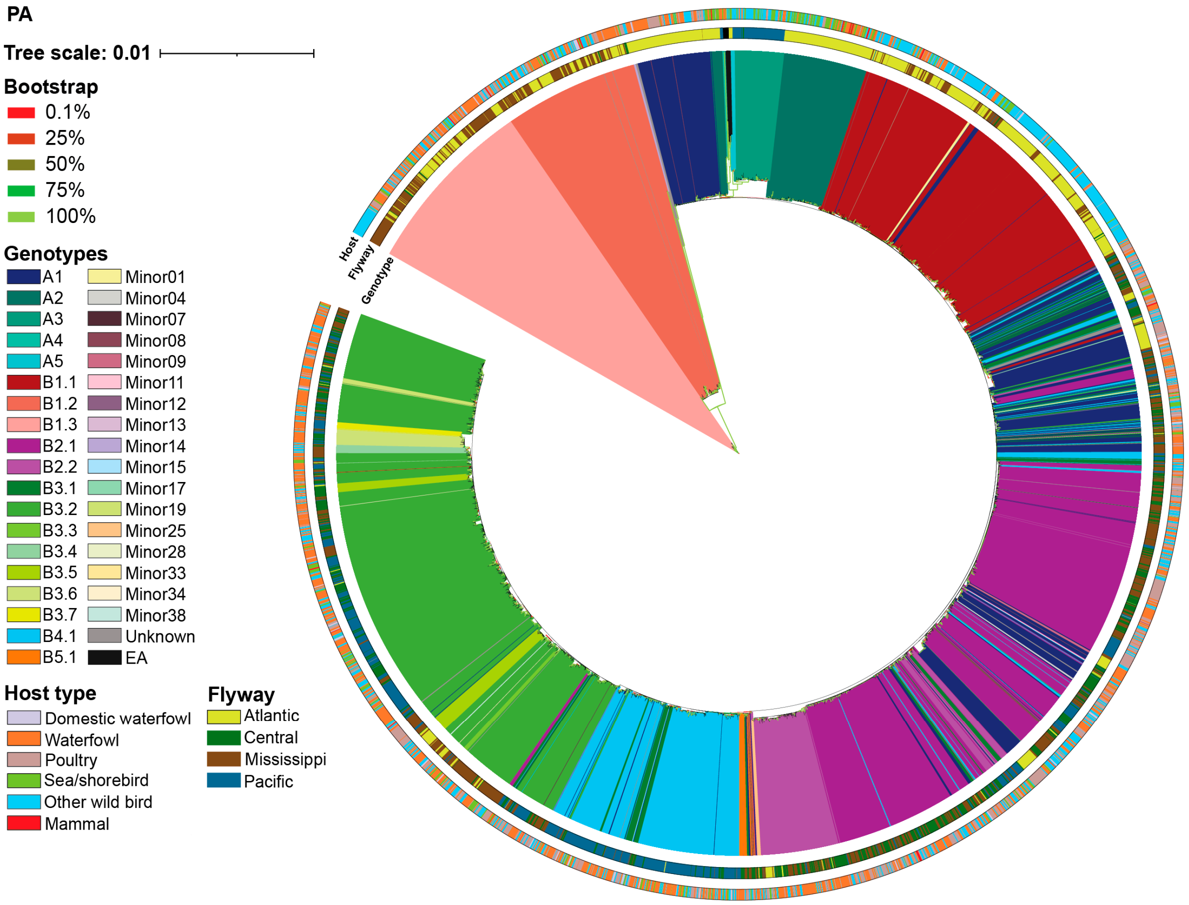

Figure S5. Phylogenetic tree with ultrafast bootstraps of PA gene segment. Genotypes, flyway, and host category are color coded differently and bootstrap support values are indicated categorically.

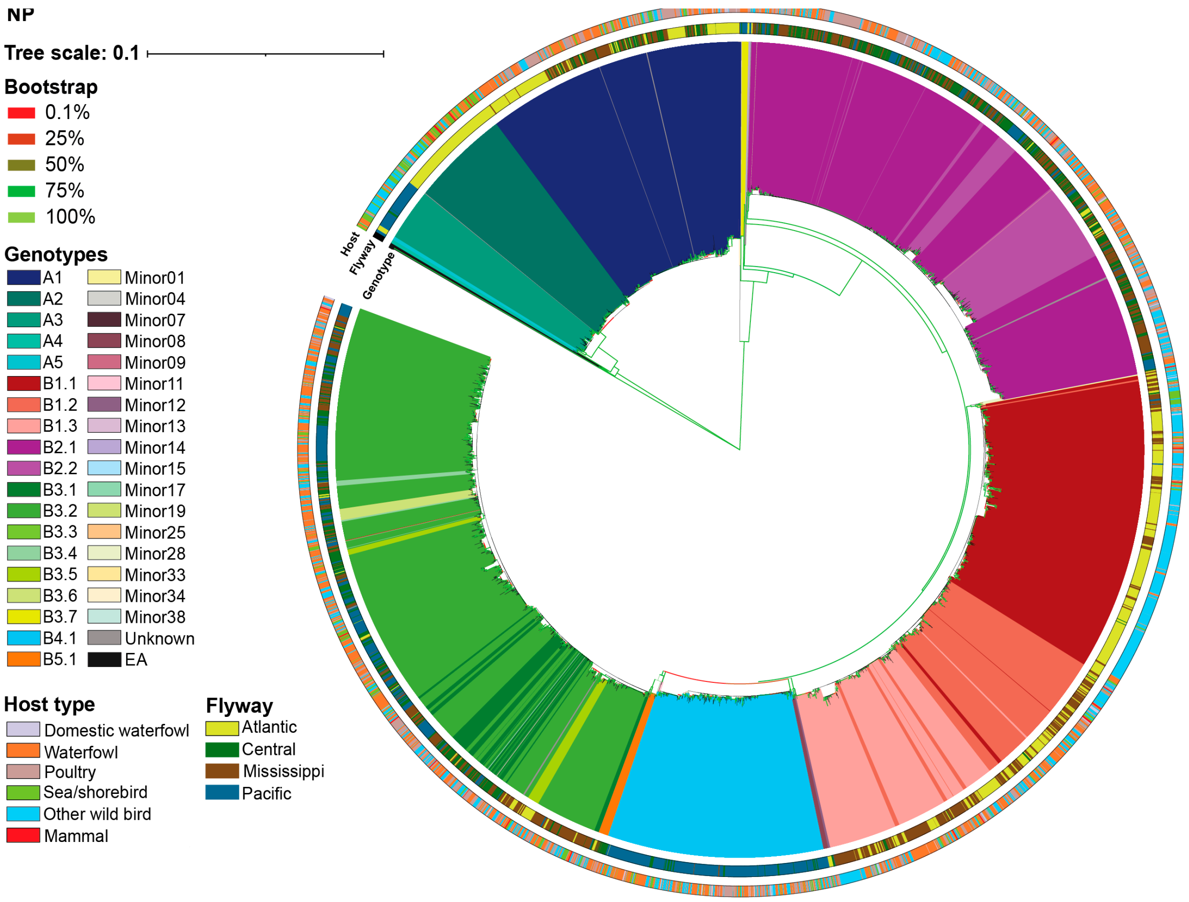

Figure S6. Phylogenetic tree with ultrafast bootstraps of NP gene segment. Genotypes, flyway, and host category are color coded differently and bootstrap support values are indicated categorically.

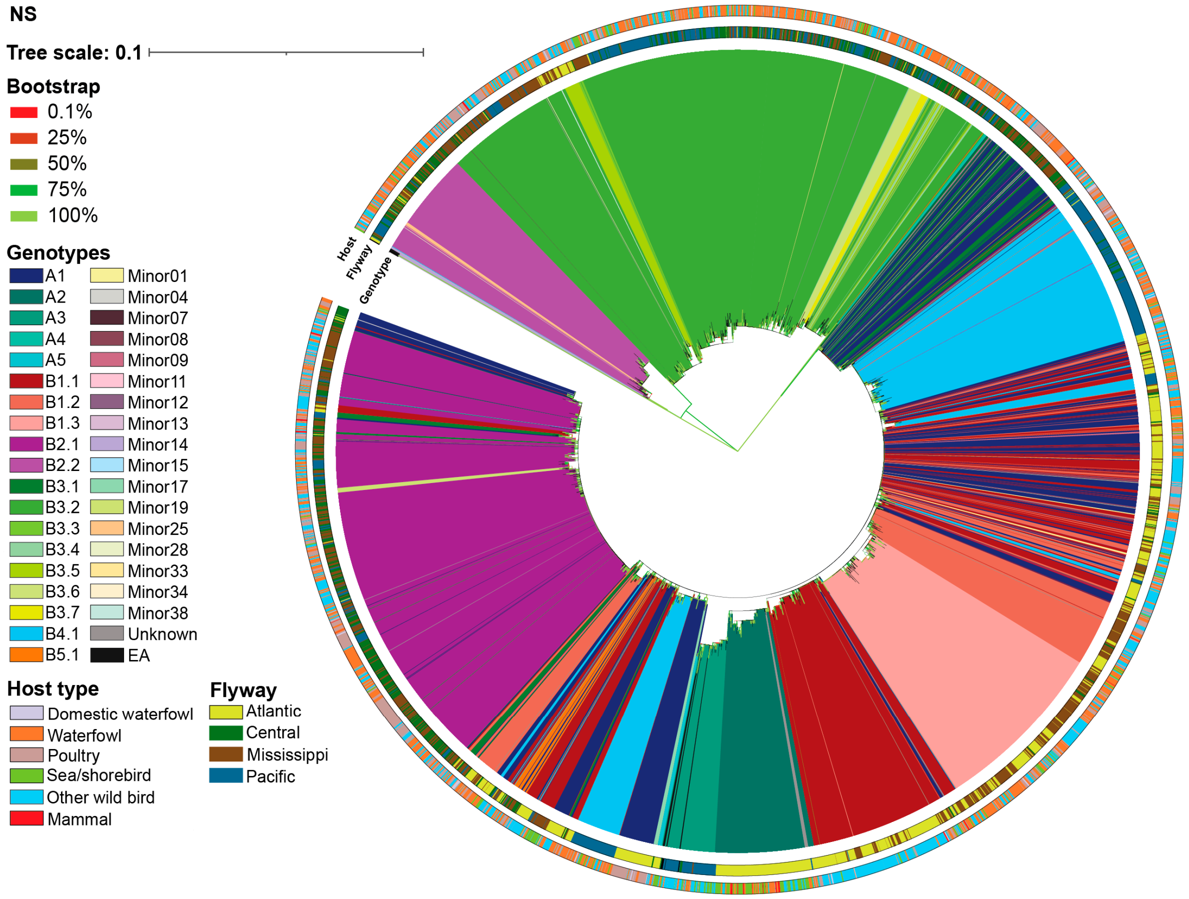

Figure S7. Phylogenetic tree with ultrafast bootstraps of NS gene segment. Genotypes, flyway, and host category are color coded differently and bootstrap support values are indicated categorically.

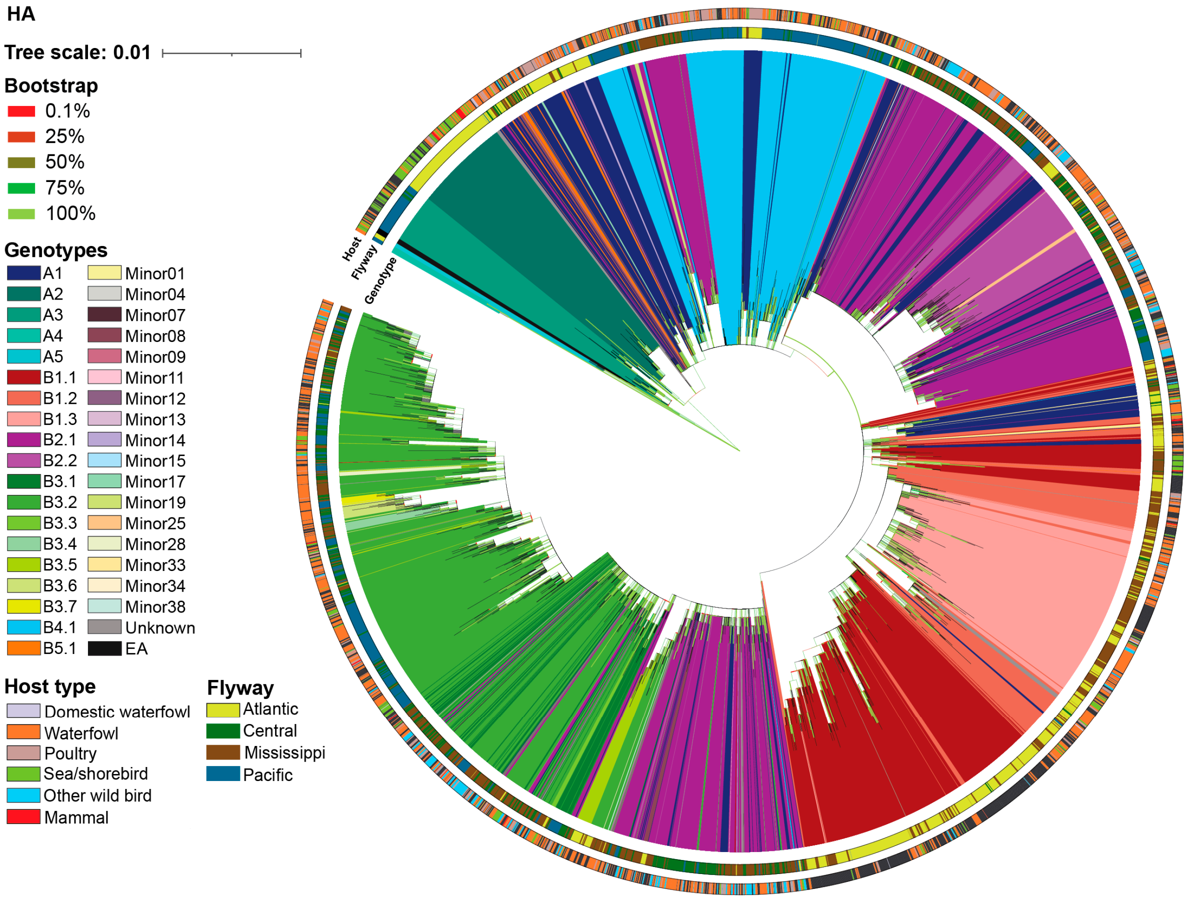

Figure S8. Phylogenetic tree with ultrafast bootstraps of HA gene segment. Genotypes, flyway, and host category are color coded differently and bootstrap support values are indicated categorically.

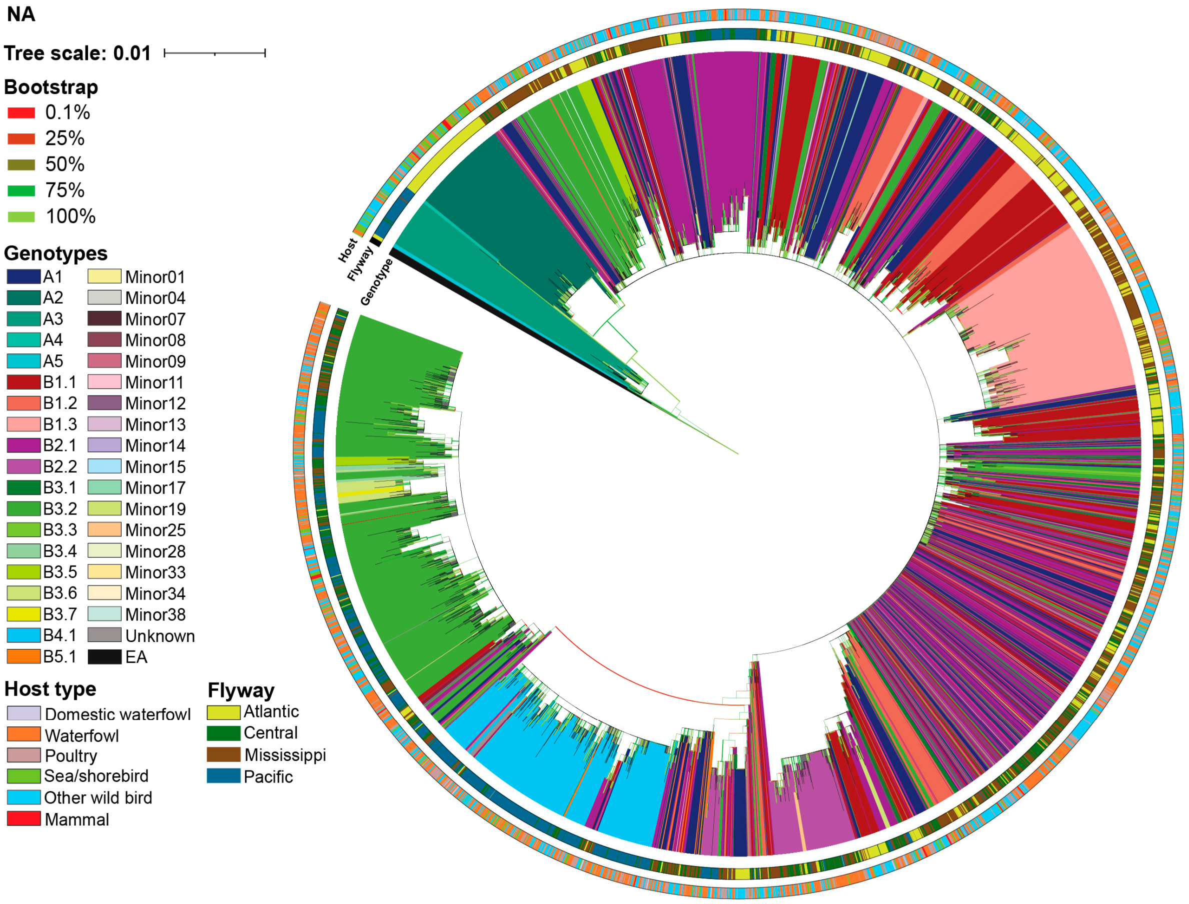

Figure S9. Phylogenetic tree with ultrafast bootstraps of NA gene segment. Genotypes, flyway, and host category are color coded differently and bootstrap support values are indicated categorically.

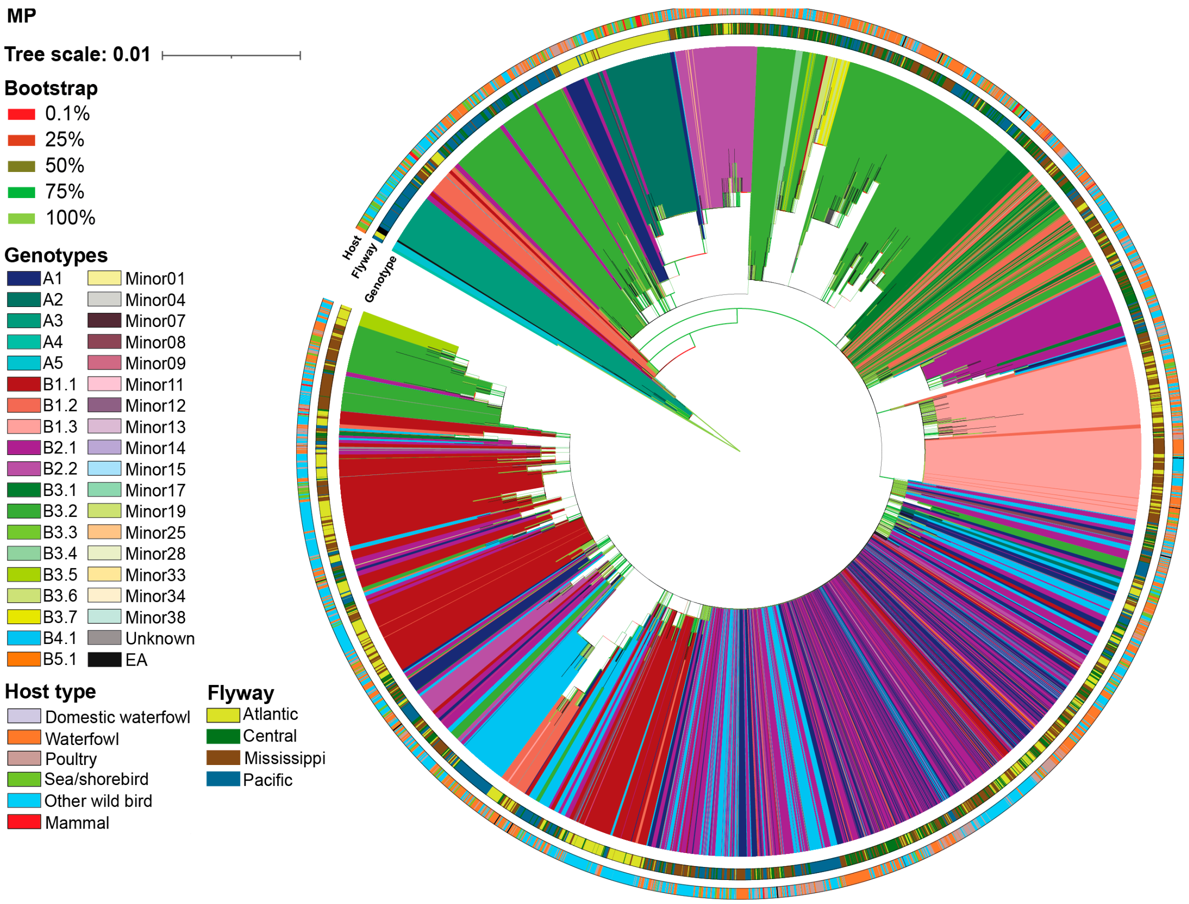

Figure S10. Phylogenetic tree with ultrafast bootstraps of MP gene segment. Genotypes, flyway, and host category are color coded differently and bootstrap support values are indicated categorically.

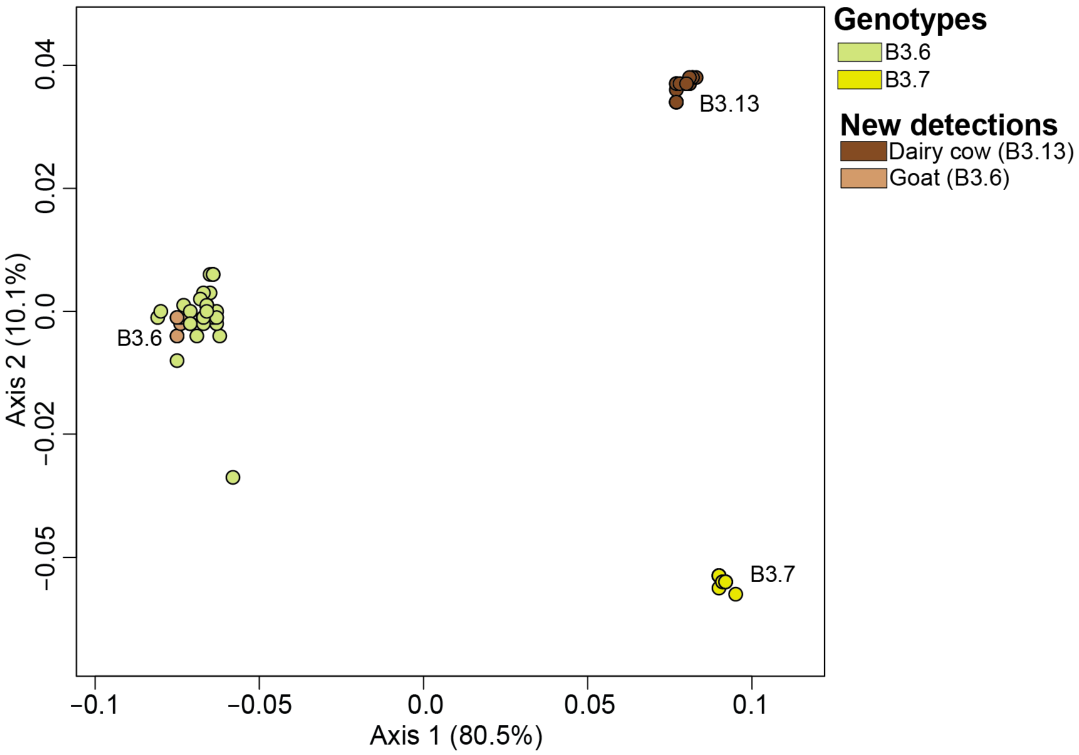

Figure S11. Multidimensional scaling to visualize genotypic diversity of reassortant H5N1 whole-genome viruses belonging to the genotypes B3.6, B3.7, and B3.13.
